# Supplementary material for: Enhancing Solubility and Dissolution Rate of Antifungal Drug Ketoconazole through Crystal Engineering
Source: Pharmaceuticals (Basel). 2023 Sep 25;16(10):1349. doi: 10.3390/ph16101349 (PMC10610424; doi:10.3390/ph16101349)
Supplement: Supplementary file 1 [file pharmaceuticals-16-01349-s001.zip › pharmaceuticals-2581110-supplementary.pdf]

*Supporting information*

# Enhancing Solubility and Dissolution Rate of Antifungal Drug Ketoconazole through Crystal Engineering

Hongmei Yu,[a,#] Li Zhang,[a,#] Meiju Liu,[a] Dezhi Yang,[a] Guorong He,[b] Baoxi Zhang,[c] Ningbo Gong,*[a] Yang Lu,*[a] Guanhua Du[d]

*a Beijing Key Laboratory of Polymorphic Drugs, Institute of Materia Medica, Chinese Academy of Medical Sciences and Peking Union Medical College, Beijing 100050, China*

*b Beijing City Key Laboratory of Drug Target Identification and Drug Screening, Institute of Materia Medica, Chinese Academy of Medical Sciences and Peking Union Medical College. Beijing 100050, China.*

# These authors contributed equally to this work.

**Table of Contents**

Table S1. Hydrogen bond geometrical parameters of crystal structures.
Table S2. Crystallographic data and structure refinement details of KTZ−VNA.
Table S3. The retention time of KTZ and CCFs.
Fig. S1 Ortep diagrams of (a) KTZ−GTA; (b) KTZ−26DHB; (c) KTZ−PCA−II; (d) KTZ−35DNB.
Fig. S2 The structure of KTZ−PCA−I cocrystal.
Fig. S3 The residual density map provided by the SXRD data of (a) KTZ−GTA; (b) KTZ−26DHB; (c) KTZ−PCA−II; (d) KTZ−35DNB.
Fig. S4 The DSC thermograms of CCFs.
Fig. S5 FT−IR spectra of KTZ, CCFs, and the corresponding synthesized multi-component crystals.
Fig. S6 PXRD patterns of residual solids after (a) equilibrium solubility and (b) powder dissolution experiments.
Fig. S7 Accelerated stability results of (a) KTZ−GTA, (b) KTZ−VNA, (c) KTZ−26DHB, (d) KTZ−PCA, (e) KTZ−35DNB.

**Table S1.** Hydrogen bond geometrical parameters of crystal structures

| Interation | D···H/ Å | H···A/ Å | D···A/ Å | ∠(DHA)/ ° | Symmetry |
|---|---|---|---|---|---|
| | | | **KTZ–GTA** | | |
| $O_5$–$H_{5A}$···$N_4$ | 0.89 | 1.75 | 2.627 | 167 | intermolecular |
| $O_8$–$H_{8A}$···$O_4$ | 0.82 | 1.82 | 2.634 | 170 | [-x, 1/2+y, 3/2-z] |
| | | | **KTZ–VNA** | | |
| $O_7$–$H_7$···$O_4$ | 0.82 | 1.87 | 2.668 | 163 | [-x+3, -y+1, -z+2] |
| $O_{6\hat{}a}$–$H_{6\hat{}a}$···$O_{6\hat{}a}$ | 0.82 | 2.42 | 2.967 | 125 | [-x+2, -y+1, -z+1] |
| $O_{6A\hat{}a}$–$H_{6A\hat{}a}$···$N_4$ | 0.82 | 1.92 | 2.384 | 115 | intramolecular |
| | | | **KTZ–26DHB** | | |
| $N_4$–$H_4$···$O_6$ | 0.96 | 1.688 | 2.649 | 175 | intermolecular |
| $O_7$–$H_7$···$O_5$ | 0.82 | 1.798 | 2.517 | 146 | intramolecular |
| $O_8$–$H_8$···$O_6$ | 0.820 | 1.791 | 2.522 | 148 | intramolecular |
| | | | **KTZ–PCA-II** | | |
| $O_6$–$H_6$···$O_4$ | 0.82 | 1.81 | 2.604 | 162 | [x, y, z+1] |
| $O_7$–$H_7$···$O_8$ | 0.82 | 2.24 | 2.689 | 115 | intramolecular |
| $O_7$–$H_7$···$O_8$ | 0.82 | 2.08 | 2.778 | 144 | [-x+2, -y+2, -z+1] |
| $O_8$–$H_8$···$N_4$ | 0.82 | 1.79 | 2.595 | 169 | [-x+1, -y+2, -z+1] |
| | | | **KTZ–35DNB** | | |
| $O_6$–$H_6$···$N_4$ | 0.82 | 1.73 | 2.528 | 164 | intermolecular |
| $C_{23}$–$H_{23B}$···$O_4$ | 0.97 | 2.32 | 3.251 | 160 | [x-1, y, z-1] |

**Table S2.** Crystallographic data and structure refinement details of KTZ−VNA

| | KTZ−VNA |
|---|---|
| Empirical formula | $C_{26}H_{25}Cl_2N_4O_4$, $C_8H_8O_4$ |
| Molecule weight | 696.54 |
| Crystal size (mm) | 0.15 × 0.27 × 0.33 |
| Temperature (K) | 293 (2) |
| Description | block |
| Crystal system | triclinic |
| Space group | $P-1$ |
| $a$ (Å) | 8.236 (1) |
| $b$ (Å) | 13.995 (1) |
| $c$ (Å) | 14.869 (1) |
| $\alpha$ (°) | 86.78 (1) |
| $\beta$ (°) | 80.45 (1) |
| $\gamma$ (°) | 80.92 (1) |
| Volume (Å$^3$) | 1668.17(12) |
| $Z$ | 2 |
| Density (g cm$^{-3}$) | 1.387 |
| Independent reflections | 6357 |
| Reflections with $I > 2\ \sigma(I)$ | 4021 |
| $R_{int}$ | 0.0521 |

| | |
|---|---|
| final $R$, $wR(F^2)$ value $[I > 2\ \sigma(I)]$ | 0.222, 0.582 |
| Goodness-of-fit on $F^2$ | 2.414 |

**Table S3.** The retention time of KTZ and CCFs

| Sample | KTZ | GTA | VNA | 26DHB | PCA | 35DNB |
|---|---|---|---|---|---|---|
| Retention time | 5.77 | / | 1.92 | 1.88 | 2.17 | 1.95 |

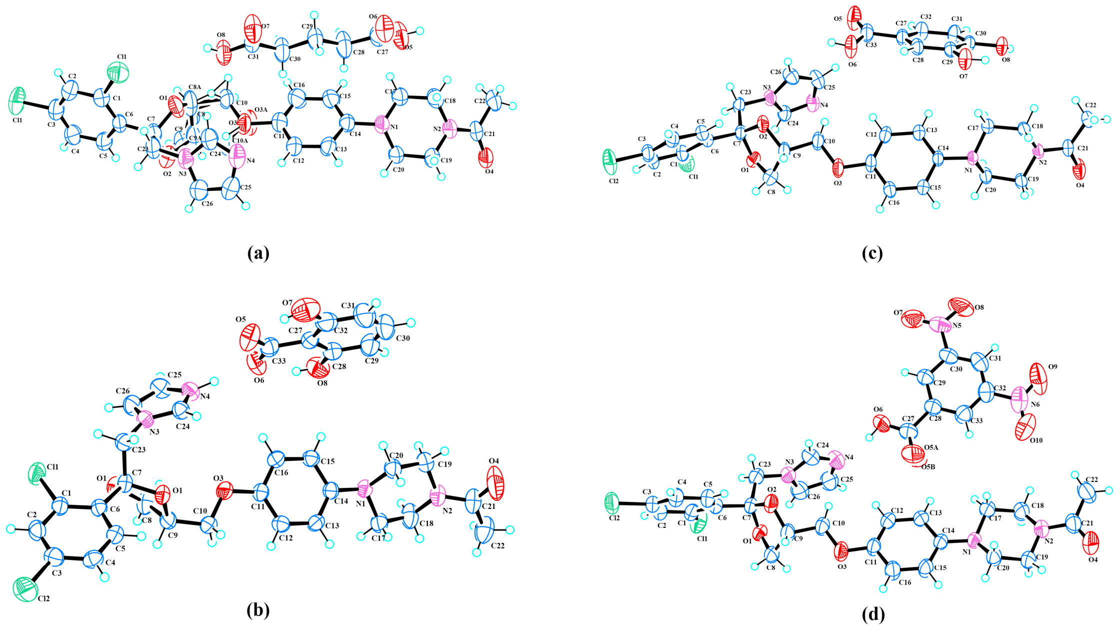


**Fig. S1** Ortep diagrams of (a) KTZ−GTA; (b) KTZ−26DHB; (c) KTZ−PCA−II; (d) KTZ−35DNB.

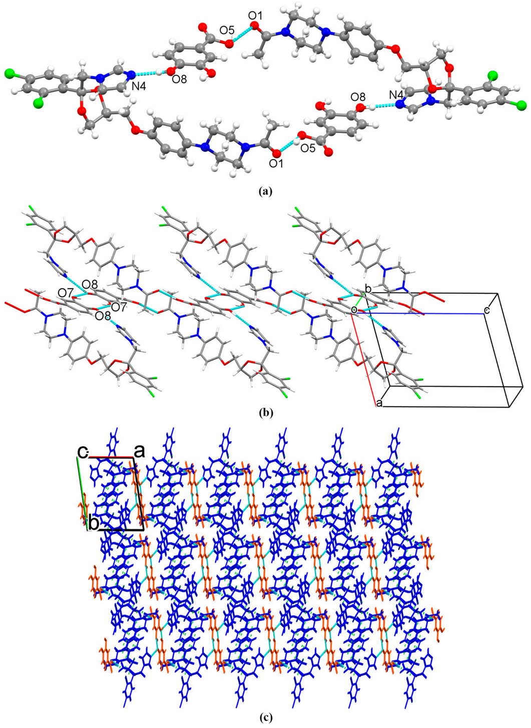


**Fig. S2** (a) Hydrogen-bonding interactions in an annular tetrameric structure; (b) the annular tetramers propagate to form a chain structure; (c) the crystal packing diagram viewed along the crystallographic c-axis.

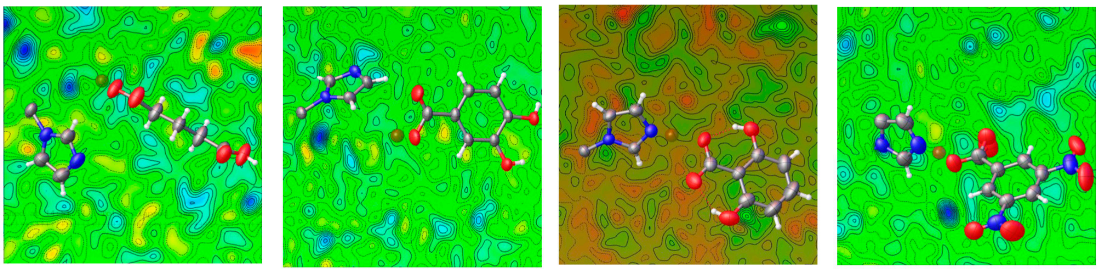

(a) (b) (c) (d)

**Fig. S3** The residual density map provided by the SXRD data of (a) KTZ−GTA; (b) KTZ−26DHB; (c) KTZ−PCA−II; (d) KTZ−35DNB.

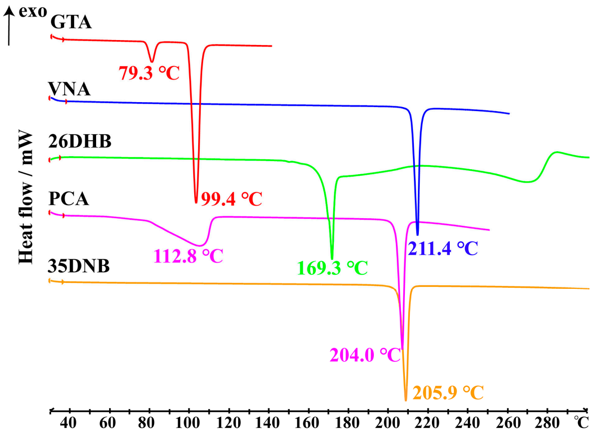


**Fig. S4** The DSC thermograms of CCFs used in this study.

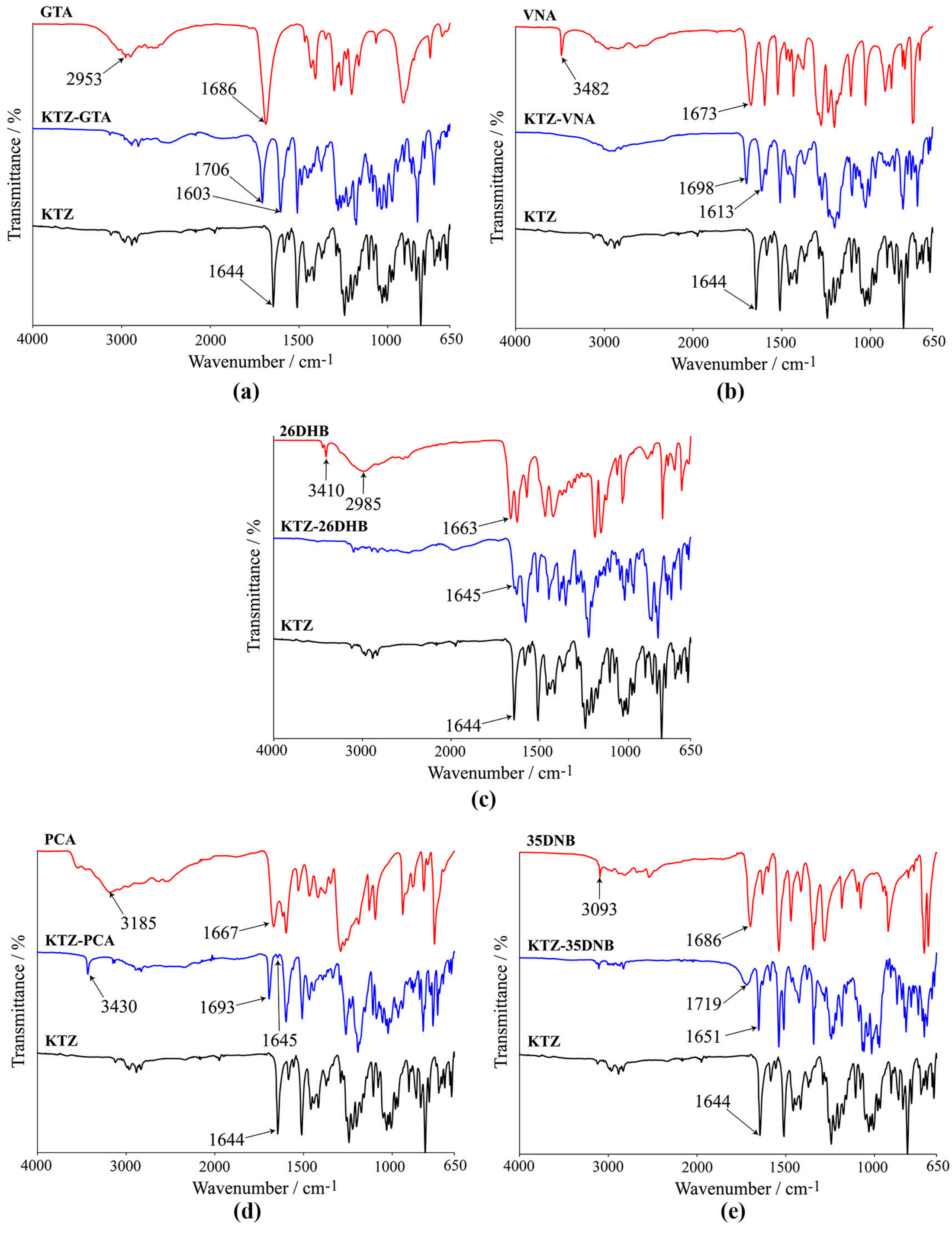


**Fig. S5** FT−IR spectra of KTZ, CCFs, and the corresponding synthesized multi-component crystals.

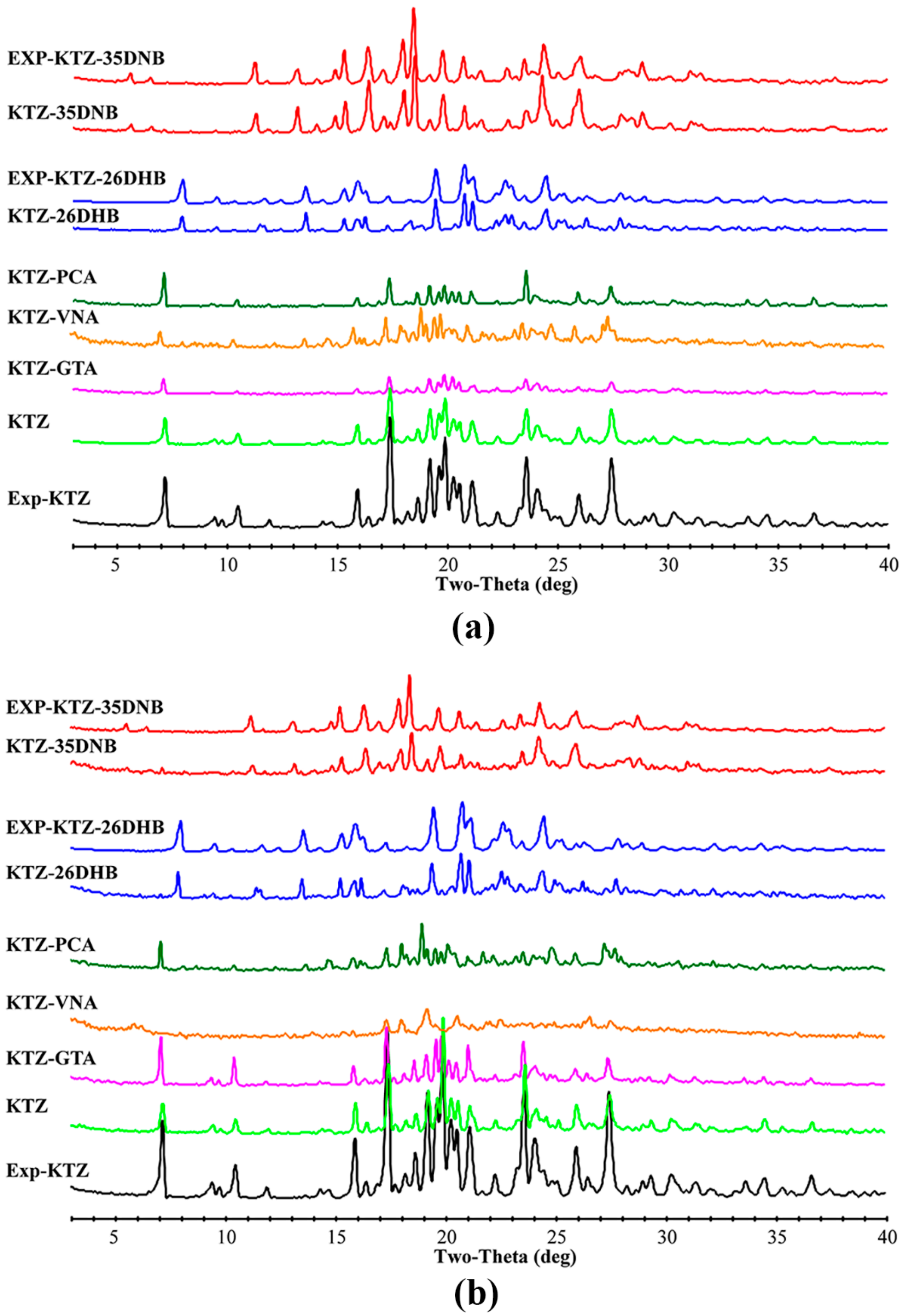


**Fig. S6** PXRD patterns of residual solids after (a) equilibrium solubility and (b) powder dissolution experiments.

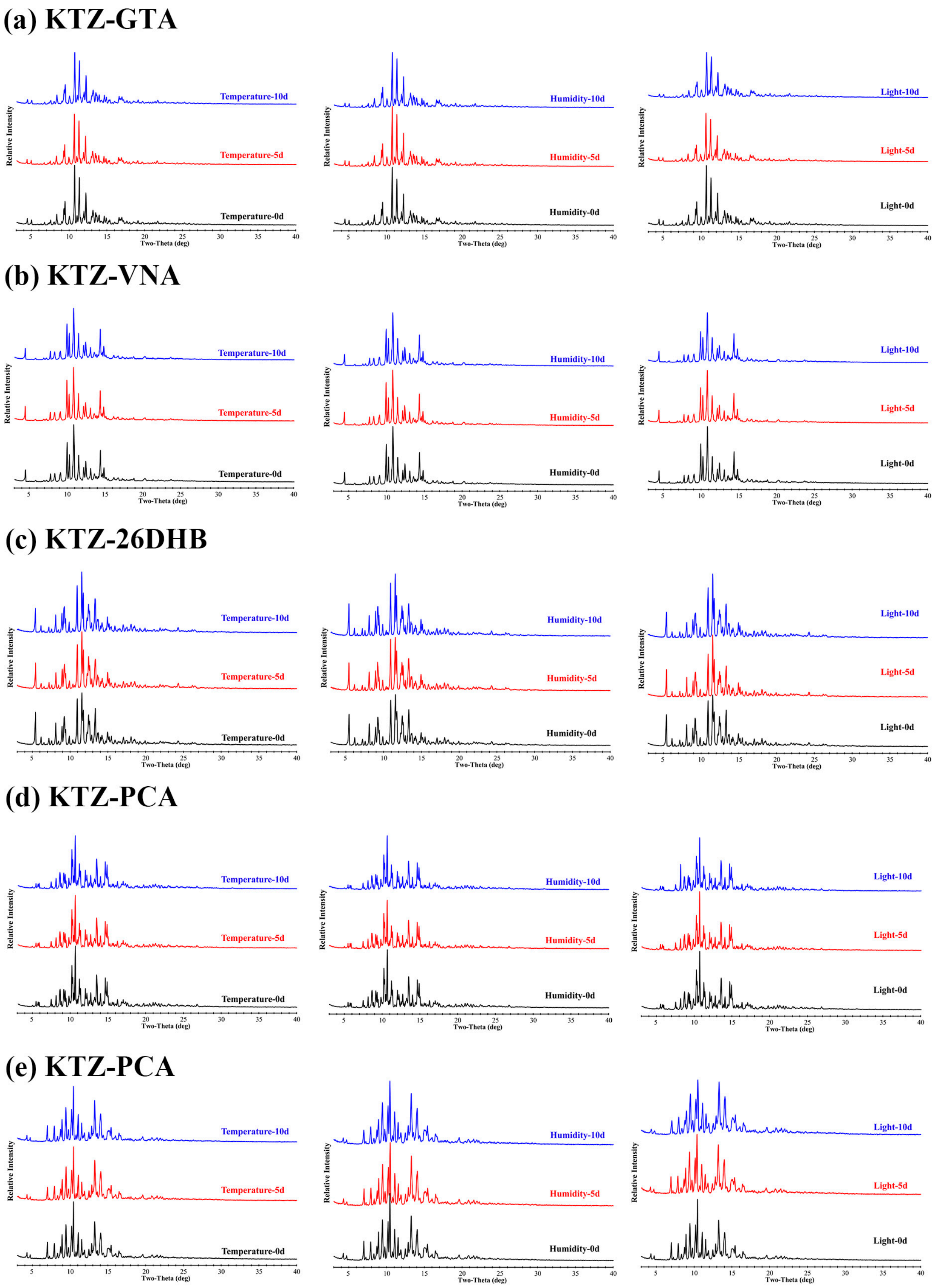


**Fig. S7** Accelerated stability results of (a) KTZ−GTA, (b) KTZ−VNA, (c) KTZ−26DHB, (d) KTZ−PCA, (e) KTZ−35DNB.
